# Development of the multivariate administrative data cystectomy model and its impact on misclassification bias

**DOI:** 10.1186/s12874-024-02199-1

**Published:** 2024-03-21

**Authors:** James Ross, Luke T. Lavallee, Duane Hickling, Carl van Walraven

**Affiliations:** 1 Department of Surgery, University of Ottawa, Ottawa, Canada; 2 Department of Medicine / Department of Epidemiology & Community Medicine, University of Ottawa, ASB1-003, 1053 Carling Ave, Ottawa, ON K1Y 4E9 Canada; 3 Ottawa Hospital Research Institute, ASB1-003, 1053 Carling Ave, Ottawa, ON K1Y 4E9 Canada; 4 ICES-uOttawa, ASB1-003, 1053 Carling Ave, Ottawa, ON K1Y 4E9 Canada

**Keywords:** Cystectomy, Urinary diversion, Predictive model, Misclassification bias, Bootstrap imputation, Administrative data

## Abstract

**Background:**

Misclassification bias (MB) is the deviation of measured from true values due to incorrect case assignment. This study compared MB when cystectomy status was determined using administrative database codes vs. predicted cystectomy probability.

**Methods:**

We identified every primary cystectomy-diversion type at a single hospital 2009–2019. We linked to claims data to measure true association of cystectomy with 30 patient and hospitalization factors. Associations were also measured when cystectomy status was assigned using billing codes and by cystectomy probability from multivariate logistic regression model with covariates from administrative data. MB was the difference between measured and true associations.

**Results:**

500 people underwent cystectomy (0.12% of 428 677 hospitalizations). Sensitivity and positive predictive values for cystectomy codes were 97.1% and 58.6% for incontinent diversions and 100.0% and 48.4% for continent diversions, respectively. The model accurately predicted cystectomy-incontinent diversion (c-statistic [C] 0.999, Integrated Calibration Index [ICI] 0.000) and cystectomy-continent diversion (C:1.000, ICI 0.000) probabilities. MB was significantly lower when model-based predictions was used to impute cystectomy-diversion type status using for both incontinent cystectomy (F = 12.75; *p* < .0001) and continent cystectomy (F = 11.25; *p* < .0001).

**Conclusions:**

A model using administrative data accurately returned the probability that cystectomy by diversion type occurred during a hospitalization. Using this model to impute cystectomy status minimized MB. Accuracy of administrative database research can be increased by using probabilistic imputation to determine case status instead of individual codes.

**Supplementary Information:**

The online version contains supplementary material available at 10.1186/s12874-024-02199-1.

## What is new

**Key Findings:** A model that exclusively used administrative data accurately to predict the probability that cystectomy by diversion type occurred during a hospitalization was very accurate. Using this model to impute cystectomy status minimized misclassification bias compared to using procedure codes.

**What this adds to what is known:** Using codes in health administration data to classify patients regarding procedure status can cause erroneous results. Using an accurate multivariate model to impute procedure status can reduce this bias.

**What should change now:** Researchers using health administrative data should quantify the accuracy of codes used to determine primary exposure status and consider using accurate multivariate models for exposure status imputation.

## Introduction

Health administrative data (HAD) record information required for regulatory or financial purposes in health care systems. Most HAD-based research uses codes to identify diagnoses or procedures. However, these codes arenever perfectly accurate, and their use returns results that deviate from true values due to incorrect case status assignment. This deviation of measured from true values is termed “misclassification bias” [1]. Misclassification independent of other variables is termed ‘non-differential’ and will bias association measures towards the null; misclassification that varies by other variables is termed ‘differential’ and can bias association measures with those variables in any direction. All misclassification can bias prevalence estimates in unpredictable directions. Despite it being widely known that codes can be erroneously applied, HAD-based studies often identify cases or outcomes using codes or code combinations with unknown accuracy [2].

HAD are appealing for studying cystectomy since this is a relatively uncommon procedure with notable process variation having considerable morbidity and mortality. Cystectomy with urinary diversion is the standard surgical treatment for both muscle invasive and high-risk non-muscle invasive bladder cancer. Following bladder removal, urinary diversion must be performed to maintain continuous urine drainage and preserve renal function. Urinary diversion is commonly performed using a segment of intestine to which the ureters are attached. This urinary diversion can be either continent or incontinent. A continent diversion usually includes a “neobladder” in which a segment of intestine is reconfigured in a spherical fashion and placed in the bladder’s usual location that is attached to the urethra. Therefore, patients store urine in the new bladder and then voluntarily pass urine when able. If this is not possible, a continent cutaneous diversion is created wherein a “catheterizable” pouch is formed by attaching the neobladder to the abdominal wall with a continent stoma through which patients can insert a catheter to drain the stored urine. An incontinent diversion includes a urinary conduit created by attaching a segment of intestine to the abdominal wall which continuously drains into an adherent stoma appliance. Less commonly, the ureters can be attached directly to the abdominal wall to allow for continuous drainage (i.e. cutaneous ureterostomy).

Cystectomies are usually identified in HAD by diagnostic and procedural codes. Two studies have found that a diagnostic code for bladder cancer and a procedure code for radical cystectomy was predictive of radical cystectomy for cancer: Tan *et. al* [3]. found that this combination had a sensitivity of 98.8% (95%CI 93.5–100.0) and a positive predictive value of 93.3% (85.9%-97.5); in an internal validation cohort, Lyon et. al [4]. measured a sensitivity of 97.0% (95%CI 93.9–98.8) and positive predictive value of 95.0% (91.4–97.4). However, neither of these code algorithms identified non-oncological radical cystectomy, was externally validated, or measured misclassification bias when cystectomy status was determined using these codes. In addition, these code algorithms did not distinguish between cystectomies with incontinent or continent diversions. This is important since cystectomy with continent diversion is more surgically complicated and has unique outcomes.

We were skeptical that cystectomies with different diversion types could be accurately distinguished in HAD with codes since these procedures are very similar. However, several studies have shown that multivariable prediction models using variables from HAD can accurately predict the probability of particular diagnoses or procedures [5–8]. These studies have also found that using these predicted probabilities to impute case status reduces misclassification bias compared to using codes alone.

In this study, we identified every person who had a cystectomy as a primary surgery for any indication at our hospital over a 10-year period. We then measured the association of cystectomy with 30 variables (reference standard). These association measures were repeated after determining cystectomy status using codes and quantified misclassification bias by compared these association measures to reference standard values. Finally, we created a HAD-based multivariable model that predicted cystectomy probability by urinary diversion type. We then used these probabilities to impute cystectomy status, measured associations of cystectomy with the 30 variables, and compared misclassification bias to that when cystectomy status was determined using codes. Finally, misclassification bias when determining cystectomy status based on codes or the model-based probability was compared.

## Methods

### Study setting

The study took place at The Ottawa Hospital (TOH), a 1000-bed teaching hospital with two campuses that is the tertiary referral and trauma center for a region of approximately 1.3 million people. Annually, TOH has more than 175,000 emergency department visits, 40,000 non-psychiatric admissions, and 50,000 surgical cases. During the study period, more than 95% of cystectomies in the region were conducted at TOH; near the end of the study period, all such procedures were performed at TOH.

### Ethics approval and consent to participate

The study was approved by the Ottawa Health Science Network Research Ethics Board (File: OHRI REB 20220112-01H). This study was a secondary data analysis; except for patient medical record review, all analyses involved deidentified data. The ethic board review waived the need to directly approach patients and retrieve consent for the study. The study period was 1 January 2009 to 1 June 2019. This period corresponded to the time that our hospital’s operation registry (used for case identification) existed.

### Health administrative datasets used for study

This study used five health administrative datasets (Table 1). The Surgical Information Management System (SIMS) dataset is a registry of all primary surgical procedures conducted at TOH. Following all operations, the surgical team enters the procedure that was performed. Completion rates for these forms are essentially 100% since hospital remuneration is a function of these data and nurses cannot close cases without these data. Data from these forms are inputted by hospital records staff into the operation registry with the primary procedure recorded by a 10-digit alpha-numeric code. The Discharge Abstract Database (DAD) is a population-based health administrative dataset that captures all Ontario hospitalizations, recording patient-based information (age, sex, pre-admission diagnostic codes [using the International Classification of Disease-10th Revision, ICD-10]) and hospitalization information (including admission service, procedural codes [using the Canadian Classification of Interventions, CCI] with dates, admission and discharge dates). The Ontario Health Insurance Plan (OHIP) database contains almost all surgeon service claims (by which surgeons are remunerated) that record the date and procedure type. The Ontario Cancer Registry (OCR) records the date and type of all index cancers diagnosed in Ontario. The Registered Persons Database (RPDB) records the death date of all Ontarians. DAD, OHIP, OCR, and RPDB are stored at ICES (formerly known as the Institute for Clinical Evaluative Sciences). ICES is an independent non-profit organization that houses population-based collections of health administrative datasets for the province of Ontario.

**Table 1:** Description of datasets used for study

NAME	CONTENTS	UNIT OF ANALYSIS	USE IN STUDY
**SIMS (**Surgical Information Management System**)**	All primary surgical procedures at study hospital	Procedure	Identify all potential cystectomies during study period
**DAD (**Discharge Abstract Database**)**	All hospitalizations in Ontario	Hospitalization	Create all covariates for Administrative Data Cystectomy Model
**OHIP (**Ontario Health Insurance Plan**)**	All health care claims	Procedure	Determine cystectomy claim status for each hospitalization
**OCR (**Ontario Cancer Registry**)**	All Ontario primary cancers	Case	Determine bladder cancer status
**RPDB (**Registered Persons Database**)**	Demographic information of all Ontarians	Person	Death status within 28 days of hospital admission

Each of these datasets were linked deterministically using encrypted patient identifier

### Case identification

Our first goal was to identify all cystectomies performed at TOH during the study period. This was done by querying SIMS for all potential primary cystectomy and urinary diversion procedures using the codes listed in Appendix A. To ensure complete capture of cystectomies, procedure codes for surgeries under which true cystectomies might be misclassified – such as partial cystectomy without urinary diversion or nephroureterectomy – were also included in the query (Appendix A).

True cystectomy-urinary diversion status of these potential cases were determined using manual chart review by a single reviewer (JR). This was determined by reviewing the operative note on the surgical date recorded in SIMS. If an operative note was incomplete, unclear, or missing, supplemental review of associated progress notes and discharge summary was performed. Patients who were classified with cystectomy were subclassifed with either continent or incontinent urinary diversion. The few patients who underwent cystectomy alone without urinary diversion (because both kidneys were simultaneously removed or the patient was already on dialysis for renal failure and anuric) were classified with incontinent diversion. Any case that was unclear regarding its cystectomy-urinary diversion status was reviewed with a second expert reviewer (LL) to provide a final opinion; this occurred in two cases.

We excluded patients less than 18 years of age and cases where cystectomy-urinary diversion was a secondary procedure and part of a larger surgery (such as a total pelvic exoneration for locally invasive rectal cancer). The latter cases were very uncommon and were excluded since they represent a patient cohort that was distinct from those having primary cystectomy-urinary diversion. Finally, patients without valid Ontario health card numbers were also excluded since they could not be linked to Ontario health data for analysis.

These steps identified all primary cystectomy cases at TOH during the study period; therefore, any TOH hospitalization that was *not* included in this group *did not* have a primary cystectomy. For all cystectomy cases, we recorded the: 1) Ontario health card number, 2) cystectomy date, and 3) diversion type (incontinent vs. continent). This reference cystectomy dataset was transferred to ICES via a encrypted data portal where the health card number was encrypted to permit linkage with ICES datasets.

### Creating the Administrative Data Cystectomy Model (ADCM)

We created our study’s analytical dataset by retrieving from the DAD all adult TOH hospitalizations during the study period. This dataset was linked to our reference cystectomy dataset via encrypted health card number and admission date to determine which TOH admissions truly had a cystectomy and, if so, its diversion type.

We reviewed CCI coding manuals to identify all CCI cystectomy codes (Appendix B). CCI codes used to identify cystectomy by urinary diversion within the DAD were reviewed with a Health Records expert at TOH to ensure completeness. Hospitalizations were classified as ‘coded with cystectomy by diversion type’ if they were assigned at least one CCI procedure code within the DAD (during the hospitalization) *or* at least one Ontario Health Insurance Plan (OHIP) billing code (with the service date being equal to the true operative date) for the cystectomy-diversion type.

We then reviewed the other co-variables in the DAD, OHIP database, and the OCR to identify patient-, hospital-, and procedure-level factors that might be associated with true cystectomy status. These potential covariates were ranked independently by two surgeons (JR, LL) based on their potential ability to identify cystectomy using health administrative data. These ranks were averaged to return the final covariate priority ranking (Appendix C).

To create the ADCM, multivariable multinomial logistic regression was used to model patient cystectomy-diversion type status. We used the methods proposed by Riley et. al [9]. to determine the number of degrees of freedom that our model could contain. This calculation assumed that there would be 429,000 admissions to TOH during the study period with an estimated 250 cystectomies of each diversion type, giving a prevalence of each cystectomy-diversion type of 0.0583%. We then calculated the number of degrees of freedom (*df*) permitted in the regression model given four criteria: 10 outcomes per df (25 *df* permitted); mean error around individual predictions of ± 0.05% (42.6 *df* permitted); target shrinkage of 99.9% (32.1 *df* permitted); and target optimism in model fit of 0.0005 (106 *df* permitted). The final allowable degrees of freedom in the model for each cystectomy-diversion type was the minimum of these calculations (25 *df*).

The outcome for the ADCM was true cystectomy-diversion type status and it had three values: cystectomy-incontinent diversion, cystectomy-continent diversion, or no cystectomy. The ADCM was constructed by adding covariates in rank order of perceived importance for cystectomy identification (Appendix C). Variables were retained if the likelihood ratio test following variable addition was significant at a *p*-value ≤ 0.05. We used a SAS macro described by Sauerbrei et. al [10]. to identify best single fractional polynomial transformations for continuous candidate variables (age, acute length of stay, and operative time). Model building ended when all candidate variables (Appendix C) had been offered to the model. Creation and performance of the ADCM was reported using methods suggested by the TRIPOD statement (Appendix E).

### Analysis

Model performance was internally validated using optimism-corrected c-statistic (for discrimination) and optimism-corrected integrated calibration index (ICI—for calibration) [11] using methods described by Steyerberg [12] with 1000 bootstrap samples.

To quantify misclassification bias, we first used the reference standard cystectomy-diversion type status to calculate true values of 30 statistics: cystectomy-diversion type prevalence in study cohort; the association of cystectomy with 3 continuous covariables [patient age, operation time, acute hospital length of stay] measured using linear regression; and the association of cystectomy with 27 binary covariables [sex, admission urgency, general anaesthetic status, transfusion status, discharge status, 28-day death or unplanned readmission status and 21 comorbidities from the Elixhauser morbidity scale] measured using logistic regression. With the exception of the Elixhauser comorbidities, these covariables did not require administrative database codes and are accurately measured in the DAD [13].

We then repeated the measurement of these 30 statistics after assigning cystectomy-diversion status using three methods:

- 1. *CCI / OHIP Code for Cystectomy*: Patients with a CCI or OHIP procedure code for cystectomy (Appendix B) were classified with cystectomy-diversion by code.
- 2.* ADCM-Categorical:*Youden’s method was used to determine the ADCM-based predicted probability of cystectomy by diversion type that optimized classification accuracy [14]. This threshold corresponds to the predicted cystectomy-diversion type probability that is closest to the top left-hand corner of the corresponding receiver operating characteristic (ROC) curve. Patients with expected cystectomy probabilities equal to or above this threshold were classified with cystectomy-diversion by ADCM-categorical.
- 3.* ADCM-Bootstrap Imputation:*This method used the ADCM predicted cystectomy probability to impute cystectomy status using bootstrap imputation (BI) [6–8, 15]. BI started by creating 1000 random bootstrap samples (with replacement) of the study cohort with each having a sample size identical to the original cohort. For each hospitalization within each bootstrap sample, a uniformly distributed number between 0 and 1 was randomly selected; cystectomy-diversion status was then imputed to be present if the random number was *below* the ADCM-based predicted cystectomy-diversion type probability for that patient. Within each bootstrap sample, we measured all 30 statistics; the mean value of all 1000 bootstrap samples was used as the final BI point estimate and the 2.5th and 97.5th percentiles as the confidence intervals.

We quantified misclassification bias for each of these three methods to assign cystectomy-diversion status using the standardized mean squared error (SMSE):

$$SMSE= \left(\frac{{(\beta -{\beta }_{T})}^{2}}{{\beta }_{T}}\right)$$

where: *β* is the parameter estimate of the covariable’s association with cystectomy-diversion determined by the cystectomy-diversion status assignment method (CCI/OHIP code for cystectomy, ADCM-categorical, or ADCM-BI); and ${\beta }_{T}$ is the parameter estimate of the covariable’s true association with cystectomy-diversion. We compared misclassification bias between cystectomy-diversion status assignment methods using ANOVA on log transformed SMSEs of all 30 variables. Differences between assignment methods was determined using Tukey’s studentized range test for ANOVA. All analyses were conducted using SAS 9.4.

## Results

**Fig. 1:**
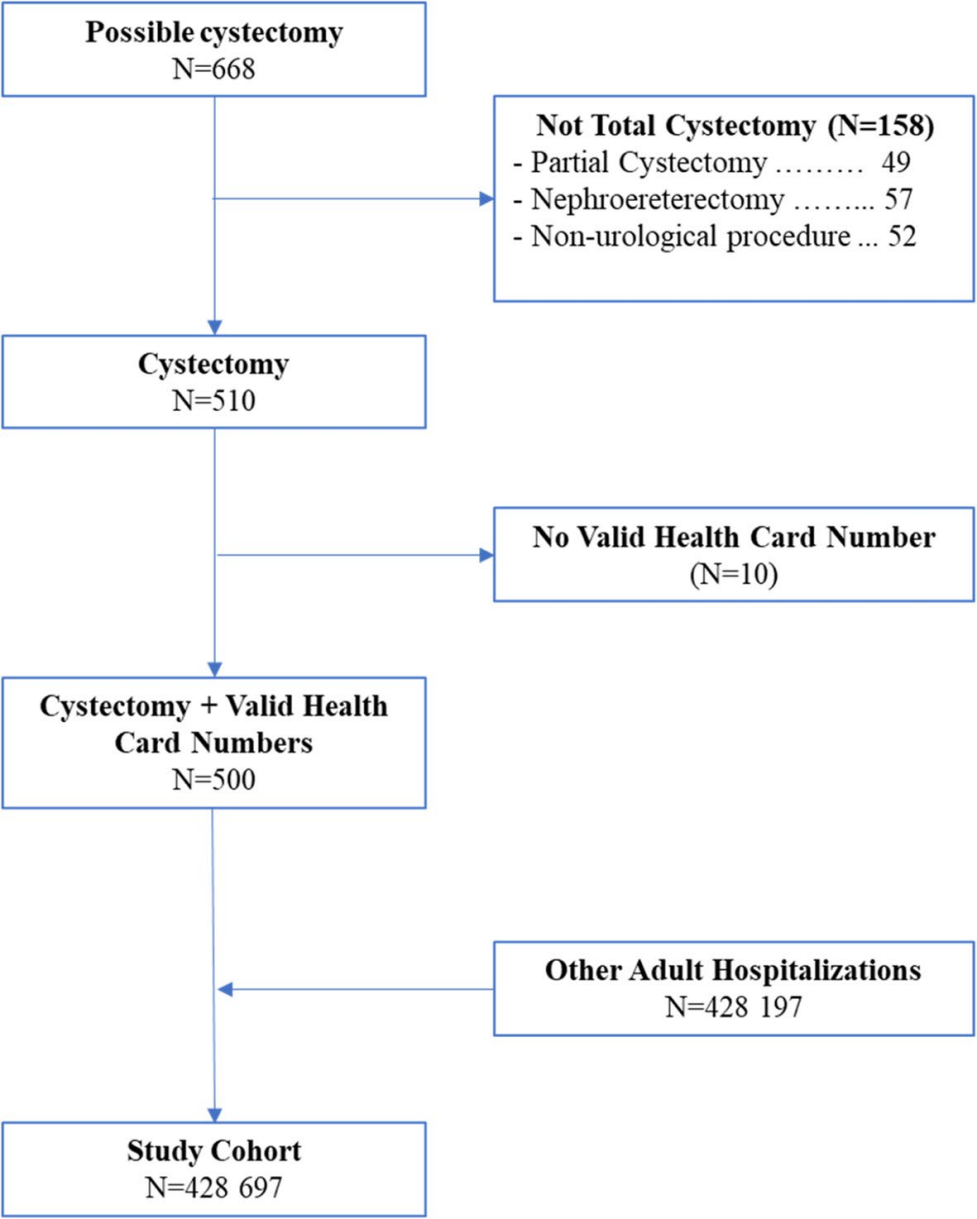
Creation of study cohort

Query of the hospital’s surgical database identified 668 possible total cystectomies (Fig. 1). Medical record review found that 158 (23.6%) were another procedure. Ten total cystectomies (2.0%) were excluded because patients did not have a valid health card number. This left 500 cystectomies in the study, to which 428 197 other adult hospitalizations during the study period were added.

The final cohort included 428 697 hospitalizations (Table 2). Patients were middle-aged (mean age 57.5 years) with the majority being female. A previous diagnosis of bladder cancer in the Ontario Cancer Registry or the Discharge Abstract Database was present in 1.5% and 0.7% of hospitalizations, respectively. A third of hospitalizations were elective and 5.0% were admitted under urology. Almost two thirds of patients (62.3%) were discharged home without supports. In less than 10% of hospitalizations did patients die or get urgently readmitted to any hospital in the month after discharge.

**Table 2:** Study Cohort

	**Cystectomy-Diversion Type**		**No Cystectomy (*n* = 428 197)**	**Overall (*n* = 428 697)**
	**Incontinent (*n* = 278)**	**Continent (*n* = 222)**		
***PATIENT***				
Mean Age (SD)^a^	71.2 (10.4)	63.6 (8.5)	57.5 (20.6)	57.5 (20.6)
Male (%)^a^	201 (72.3)	177 (79.7)	173 465 (40.5)	173 843 (40.6)
Bladder Cancer—OCR^b^	218 (78.8)	204 (91.9)	6 112 (1.4)	6 535 (1.5)
- DAD^a^	234 (84.2)	218 (98.2)	2 732 (0.6)	3 184 (0.7)
***HOSPITALIZATION***				
Elective Admission (%)^a^	265 (95.3)	218 (98.2)	143 112 (33.4)	143 595 (33.5)
Urology 1° Service (%)^a^	276 (99.3)	222 (100.0)	20 846 (4.9)	21 344 (5.0)
Blood Transfusion (%)^a^	165 (59.4)	122 (55.0)	53 547 (12.5)	53 834 (12.6)
***PROCEDURAL***				
Cystectomy Code—Incontinent (%)^a^	270 (97.1)	23 (28.4)	128 (0.0)	461 (0.1)
- Continent (%)^a^	150 (54.0)	222 (100.0)	87 (0.0)	459 (0.1)
1° procedure—In Main OR^a^	275 (98.9)	222 (100.0)	145 473 (34.0)	145 970 (34.0)
- Not in Main OR^a^	≤ 5 (0.7)	0 (0.0)	177 6XX (41.X)	177 604 (41.4)
- None		0 (0.0)		
General Anesthetic (%)^a^	265 (95.3)	215 (96.8)	114 629 (26.8)	115 109 (26.9)
Median 1° Procedure, min (IQR)^a^	526 (468, 590)	641 (576, 695)	0.0 (0.0, 113)	0.0 (0.0, 113)
***OUTCOMES***				
Unplanned Return to OR, 28d (%)^a^	21 (7.6)	19 (8.6)	12 (0.0)	52 (0.0)
DC Home, no supports (%)^a^	36 (12.9)	48 (21.6)	266 924 (62.3)	267 008 (62.3)
Death/Readmit in 28d (%)^a,c^	25 (9.0)	19 (8.6)	37 587 (8.8)	37 631 (8.8)
Median Acute LOS (IQR)^a^	11.0 (8.0, 19.0)	10.0 (8.0–19.0)	3.0 (2.0, 7.0)	3.0 (2.0, 7.0)

The database source (Table 2) of each covariate is indicated (^a^Discharge Abstract Database, ^b^Ontario Cancer Registry, ^c^Registered Persons Database)

*SD* Standard deviation, % Percentage, *OCR* Ontario Cancer Registry, *DAD* Discharge Abstract Database, 1 Primary, *OR* Operating room, *min* minutes, *IQR* Interquartile range, *d* days, *DC* Discharge, *LOS* Length of stay

There were 500 cystectomies during the study period at The Ottawa Hospital (0.12% of all hospitalizations) with incontinent diversions (*n* = 278; 55.6% of cystectomies) being slightly more common than continent (*n* = 222; 44.4%). Table 2 indicates that hospitalizations with cystectomy-incontinent diversion and cystectomy-continent diversion (compared to hospitalizations with no cystectomy) were notably *more* likely to: have a previous diagnosis of bladder cancer recorded in either the OCR (78.8% and 91.9% vs. 1.4%, respectively) or the DAD (84.2% and 98.2% vs. 0.6%); be admitted electively (95.3% and 98.2% vs. 33.4%) or under a urological service (99.3% and 100.0% vs. 4.9%); be assigned a cystectomy-incontinent diversion (97.1% and 28.4% vs. 0.0%) or cystectomy-continent diversion (54.0% and 100.0% vs. 0.0%) code; have an unplanned return to the operating room (7.6% and 8.6% vs. 0.0%); and have longer median hospital lengths of stay (11 days and 10 days vs. 3 days). Compared to continent cystectomies, those with incontinent diversions were older (mean age 71.2 vs. 62.6 years), less likely to be previously coded with bladder cancer in OCR (78.8% vs 91.9%), were more likely to be coded with cystectomy-incontinent diversions (97.1% vs. 28.4%), and less likely to be discharged home without supports (12.9% vs. 21.6%) (Table 2).

Codes for cystectomy-incontinent diversion and cystectomy-continent diversion (Appendix B) had excellent sensitivities (97.1% and 100.0%, respectively) and specificities (100.0% and 100.0%) but poor positive predictive values (58.6% and 48.4%) (Table 3A). When diversion type was ignored, as was done in previous cystectomy code validation studies [3, 4], positive predictive value increased to 76.0% (95%CI 72.5–79.2).

**Table 3:** Cystectomy classification accuracy with codes and using Administrative Data Cystectomy Model (ADCM)

A. Cystectomy Status Using Administrative Database Codes					
		**Cystectomy-Incontinent Diversion Status**			
		** + **	**-**	**Sens**	97.1%
**Coded With Cystectomy—Incontinent Diversion**	** + **	270	191	**Spec**	100.0%
	**-**	8	428,228	** + PV**	58.6%
				**-PV**	100.0%
				** + LR**	2178.5
				**-LR**	0.0
		**Cystectomy-Continent Diversion Status**			
		** + **	**-**	**Sens**	100.0%
**Coded With Cystectomy—Continent Diversion**	** + **	222	237	**Spec**	99.4%
	**-**	0	428,238	** + PV**	48.4%
				**-PV**	100.0%
				** + LR**	1807.9
				**-LR**	0.0
B. Cystectomy Status Using ADCM and Best Expected Probability Thresholds*					
		**Cystectomy-Incontinent Diversion Status**			
		** + **	**-**	**Sens**	99.6%
**ADCM Cystectomy—Incontinent Diversion**	** + **	277	1614	**Spec**	99.6%
	**-**	1	426,805	** + PV**	14.6%
				**-PV**	100.0%
				** + LR**	264.5
				**-LR**	0.0
		**Cystectomy-Continent Diversion Status**			
		** + **	**-**	**Sens**	100.0%
**ADCM Cystectomy—Continent Diversion**	** + **	222	80	**Spec**	100.0%
	**-**	0	428,395	** + PV**	73.5%
				**-PV**	100.0%
				** + LR**	5355.9
				**-LR**	0.0

For cystectomy status classification by administrative database codes (Section A), patients were classified with cystectomy by diversion type if they met criteria listed in Appendix B. For cystectomy status classification using the ADCM (Section B), patients were classified with cystectomy-incontinent diversion if the expected probability from the ADCM was at least 0.0922%; patients were classified with cystectomy-continent diversion if the expected probability from the ADCM was at least 0.0626%. (*Sens* Sensitivity, *Spec* Specificity, + *PV* Positive predictive value, -*PV* negative predictive value, + *LR* Positive likelihood ratio, *-LR* Negative likelihood ratio). Values have been rounded to 1 decimal place. *As determined using Youden’s method

**Fig. 2:**
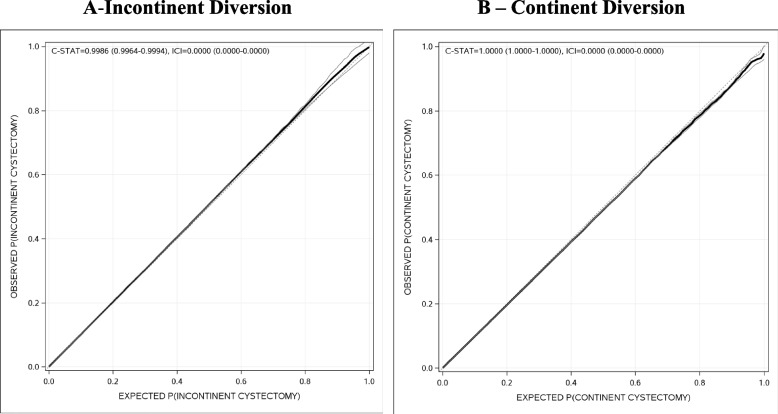
Performance of the Administrative Data Cystectomy Models. In both plots, the vertical axis indicates the observed probability of cystectomy with incontinent diversion (**A**) or continent diversion (**B**), calculated using a LOESS model. The horizontal axis presents the expected probability of cystectomy with incontinent diversion (**A**) or continent diversion (**B**), calculated using the respective Administrative Data Cystectomy Model (Appendix D). Each plot presents the model’s optimism-adjusted internally validated discrimination and calibration using the C-statistic (C-STAT) and the integrated calibration index (ICI), respectively

The Administrative Data Cystectomy Model (ADCM) included 6 variables (Appendix D). Compared to hospitalizations without cystectomy, both cystectomy types were significantly more likely to have an elective admission, get admitted under urology, or experience an unplanned return to the operating room within 28 days. Cystectomy-incontinent diversions were more likely to be coded as such by either OHIP or CCI codes and had a significantly longer hospital length of stay. Cystectomy-continent diversions were more likely to be coded as such by either OHIP or CCI codes and have a previous diagnosis of bladder cancer in DAD. The ADCM had excellent discrimination (optimism-adjusted c-statistic, incontinent diversion: 0.9986 [95%CI 0.9964–0.9994]; continent diversion: 1.0000 [95%CI 1.0000–1.0000]) and calibration (optimism-adjusted ICI of 0.0000, 95%CI 0.0000–0.0000 for both incontinent and continent diversions). Observed and expected cystectomy probabilities were very similar for both cystectomy types (Fig. 2).

By Youden’s method, the predicted cystectomy probabilities returning the most accurate classification for incontinent and continent diversions was 0.0922% and 0.0626%, respectively. Compared to codes for cystectomy-incontinent diversion (Appendix B), status assignment using this threshold increased sensitivity to 99.6% but decreased specificity from 100.0% to 99.6% and the positive predictive value to 14.6% (Table 3B). Compared to codes for cystectomy-continent diversion (Appendix B), status assignment using the ADCM-continent threshold maintained sensitivity and specificity at 100.0% while increasing positive predictive value to 73.5% (Table 3B).

**Fig. 3:**
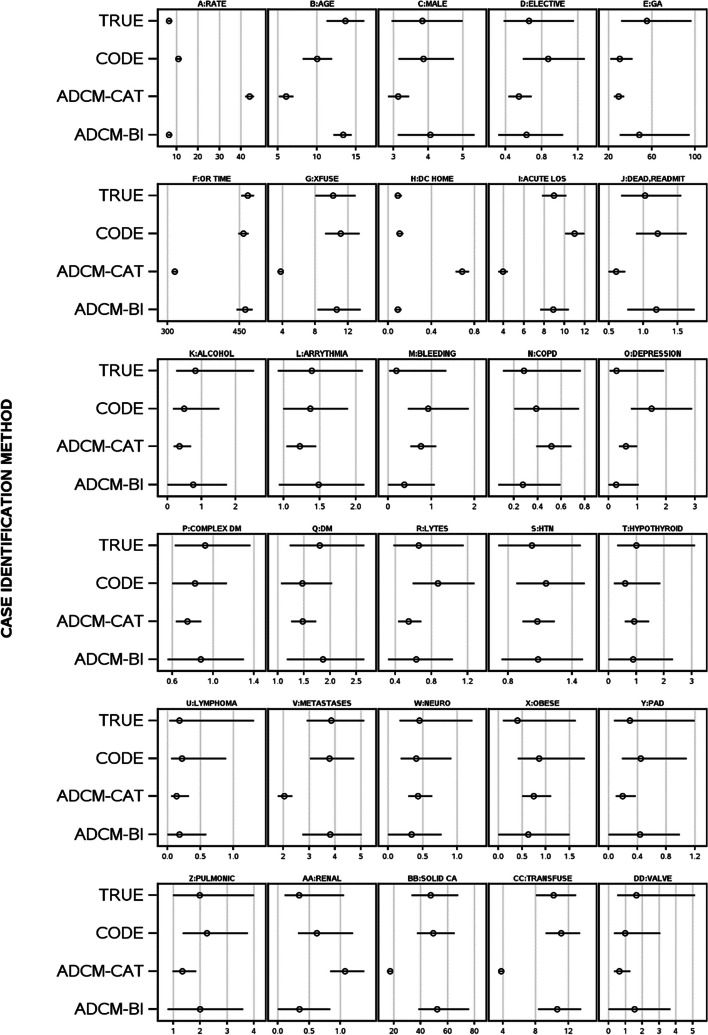
True cystectomy-incontinent diversion and its unadjusted association with other variables compared to cystectomy status determination using codes or the ADCM. Plots present cystectomy-incontinent diversion incidence (**A**) or the unadjusted association of the variable with cystectomy-incontinent diversion status determined using true status (TRUE), procedure codes in Appendix B (CODE), predicted cystectomy-incontinent probability from ADCM categorized to optimize accuracy (ADCM-CAT), or bootstrap imputation using ADCM-predicted cystectomy-incontinent probability (ADCM-BI)

Misclassification bias differed significantly when cystectomy-incontinent diversion status was determined using CCI/OHIP codes, ADCM-categorical, or ADCM-bootstrap imputation (Fig. 3A-Fig. 3DD). Procedure incidence was significantly higher when cystectomy-incontinent status was determined with CCI/OHIP-codes (+ 66.7%) or ADCM-categorical (+ 580.6%) (Fig. 3A). Estimates with ADCM-categorical returned the largest misclassification bias, being especially notable for cystectomy’s association with operation time (-32.5%, Fig. 3F), transfusion (-63.0%, Fig. 3G), home discharge status (+ 660.3%, Fig. 3H), and acute length of stay (-56.1%, F ig. 3I). Estimates for all statistics with ADCM-bootstrap were always within the 95% confidence intervals of true values. Median (interquartile range) standardized mean squared errors for code, ADCM-categorical and ADCM-bootstrap imputation was 0.163 (0.02–0.63), 0.418 (0.10–1.78), and 0.004 (0.0004–0.059), respectively. Misclassification bias differed significantly between the different cystectomy assignment methods (F = 12.75; *p* < 0.0001) with ADCM-bootstrap imputation having significantly smaller misclassification than the others (*p* < 0.05).

**Fig. 4:**
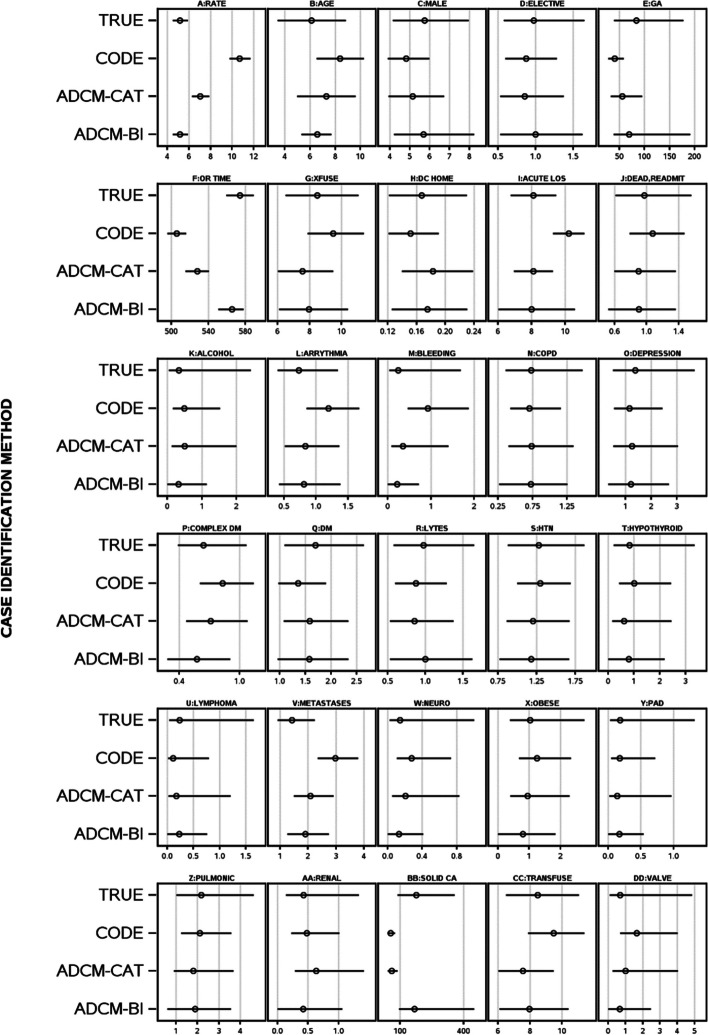
True cystectomy-continent diversion and its unadjusted association with other variables compared to cystectomy status determination using codes or the ADCM. Plots present cystectomy-continent diversion incidence (**A**) or the unadjusted association of the variable with cystectomy-continent diversion status determined using true status (TRUE), procedure codes in Appendix B (CODE), predicted cystectomy-continent probability from ADCM categorized to optimize accuracy (ADCM-CAT), or bootstrap imputation using ADCM-predicted cystectomy-continent probability (ADCM-BI)

Misclassification bias also differed significantly by cystectomy assignment methods for cystectomy-continent status (Fig. 4A-Fig. 4DD). Procedure incidence increased significantly when cystectomy-continent status was determined with CCI/OHIP-codes or ADCM-categorical (Fig. 4A). Again, ADCM-bootstrap estimates for all statistics were always within the 95% confidence intervals of true values. Median (interquartile range) standardized mean squared errors using CCI/OHIP codes, ADCM-categorical and ADCM-bootstrap imputation was 0.228 (0.02–0.77), 0.073 (0.02–0.22), and 0.006 (0.0007–0.026), respectively. Misclassification bias differed significantly (F = 11.25; *p* < 0.0001) with ADCM-bootstrap imputation having significantly lower misclassification bias than the others (*p *< 0.05).

## Discussion

This study found that cystectomy-diversion type status can be predicted with high accuracy using a multivariate model created with health administrative data (HAD). Imputing cystectomy-diversion status based on predicted probabilities from this model significantly decreased misclassification bias compared to identifying cystectomy using HAD codes alone.

Our study had several notable findings. First, we found that procedure codes for cystectomy (Appendix B) had sensitivities and specificities approaching 100% (Table 3). Given these operating characteristics, many researchers and readers would believe that misclassification bias would be minimal and would consider code status essentially equivalent to true cystectomy status. However, our data show this conclusion is incorrect since it ignores the very low prevalence of cystectomy (0.12% of all hospitalizations). As a result, these codes had low positive predictive values of 58.6% and 48.4% for incontinent and continent cystectomies, respectively (Table 3). Therefore, hospitalizations identified as having cystectomy with a particular diversion type using these codes had only about a 50% chance of truly having that procedure, resulting in notable misclassification bias (Figs. 2 and 3). These results highlight that it is essential to consider case prevalence when interpreting code accuracy measures. Second, we found that health administrative data can be used to create multivariate regression models which accurately predict procedure probability. The ADCM was extremely discriminative and well-calibrated (Fig. 2) to identify cystectomy-diversion status using health administrative data, despite the relative similarity of the two cystectomy subtypes. However, we also found that misclassification bias remained prominent when we determined cystectomy status by categorizing predicted ADCM probabilities using probability thresholds that maximized accuracy (Figs. 2 and 3). In contrast, misclassification bias was minimized when ADCM-based predictions were used to probabilistically impute cystectomy-diversion status. This phenomenon – wherein categorization of an accurate probability estimate causes misclassification bias – has been seen in previous studies [15]. These results highlight the importance of applying predicted case status using probabilistic methods like bootstrap imputation.

Several issues should be kept in mind when interpreting our results. First, although our study was done over an entire decade and included almost a half a million hospitalizations, it was completed exclusively at one center. Therefore, it would be important to repeat these methods at another hospital to ensure generalizability. Although health records abstractors – who assign diagnostic and procedural codes at each hospital in Canada – undergo similar training and follow the same coding guidelines, it is possible that coding practices might vary between hospitals. This could influence the performance of the ADCM, which is based partially on coded data. In addition, it would be important to measure the model’s performance in hospitals that are distinct from that used to derive the ADCM including non-teaching institutions and hospitals having a much lower—or even absent – incidence total cystectomy. Second, our data found that using codes to determine cystectomy status significantly increased misclassification bias compared to probabilistically imputing cystectomy status using the ADCM. However, we found no situations where cystectomy’s association with a covariate was categorically opposite when its status was determined with codes versus its true value. Some would conclude that these results validate the use of codes to determine cystectomy status since doing so never returned incorrect binary conclusions. We argue that the goal of any scientific study is to return results that are as close to the truth as possible; therefore, imputing cystectomy status using an accurate prediction model is preferable. Third, our gold standard cohort only included patients who underwent cystectomy as a primary surgery. Therefore, patients undergoing cystectomy as a secondary operation as part of a larger surgery (such as pelvic exenterations) will be excluded. These patients, however, represent a separate cohort of patients with different outcomes as compared to those who underwent cystectomy as a primary operation; we therefore feel that their exclusion is appropriate. It is also possible that a primary cystectomy was miscoded in the SIMS database (Table 1) and would have been missed in the study.

In summary, despite HAD-based procedure codes for cystectomy-diversion type having very high sensitivities and specificities, our study found that their use to impute cystectomy status resulted in important misclassification bias. This can be minimized by imputing cystectomy status using accurate cystectomy probabilities from the ADCM.

### Supplementary Information

**Supplementary Material 1. **

## Data Availability

The datasets used for this study were held securely in a linked, de-identified form and were linked using unique encoded identifiers and analyzed at ICES. While data sharing agreements prohibit ICES from making the data set publicly available, access may be granted to those who meet pre-specified criteria for confidential access, available at www.ices.on.ca/DAS.

## Abbreviations

MB: Misclassification bias
C: C-statistic
ICI: Integrated calibration index
HAD: Health administrative data
TOH: The Ottawa Hospital
DAD: Discharge Abstract Database
OHIP: Ontario Health Insurance Plan
OCR: Ontario Cancer Registry
ADCM: Administrative data cystectomy model
BI: Bootstrap imputation
