# Supplementary material for: Development of the multivariate administrative data cystectomy model and its impact on misclassification bias
Source: BMC Med Res Methodol. 2024 Mar 21;24:73. doi: 10.1186/s12874-024-02199-1 (PMC10956281; doi:10.1186/s12874-024-02199-1)
Supplement: Supplementary file 1 — Supplementary Material 1. [file 12874_2024_2199_MOESM1_ESM.docx]

**APPENDIX A:**  Codes used to query hospital’s procedure registry for cystectomies.

| **Procedure Code** | **Description** |
| --- | --- |
| **A – Codes Most Consistent with Cystectomy** | |
| BLCYSTECT | Complete removal of bladder (cystectomy) |
| BLCYSTILCD | Complete removal of bladder (cystectomy) with creation of non-continent urinary diversion (i.e. ileal conduit) |
| BLCYSTILNB | Complete removal of bladder (cystectomy) with creation of continent urinary diversion (i.e. neobladder) |
| **B – Codes That Cystectomies Might Be Misclassified With** | |
| BLCYSTECTP | Partial removal of bladder without urinary diversion |
| NEPHURETEC | Removal of kidney, ureter, and bladder cuff without urinary diversion |

**APPENDIX B:** Codes used to identify cystectomy by diversion type in health administrative data.

| **Cystectomy Type** | **CCI Code** | **OHIP Code** |
| --- | --- | --- |
| Cystectomy with Incontinent Diversion | 1PM89RR  1PM89EN  1PM89DA  1PM91LA  1PM91DA  1PM91RR  1PM91EN  1PM90LAXXG | S453  S484+S452  S484+S454 |
| Cystectomy with Continent Diversion | 1PM89LA  1PM89DAXXG  1PM89LAXXG  1PM89RRXXG  1PM89ENXXG  1PM90RDXXG  1PM91DAXXG  1PM91LAXXG  1PM91RRXXG  1PM91ENXXG  1PM92LAXXG  1PM92DAXXG  1PM92RDXXG | S440  S484+S441  S485+S441 |

Hospitalizations were classified with either cystectomy type if they were coded with either a CCI code **or** an OHIP code (with procedure date within XX weeks of hospitalization).

**APPENDIX C:** Variable creation and selection

| **Variable (Data Source)** | **Rank 1** | **Rank 2** | **Mean Rank Score** | **Final Rank** | **Cumu-lative Degrees of Freedom (DF)** |
| --- | --- | --- | --- | --- | --- |
| Billing Code for Cystectomy (OHIP) | 2 | 1 | 1.5 | 1 | 1 |
| Diagnosis of Bladder Cancer (OCR) | 3 | 2 | 2.5 | 2 | 2 |
| CCI Code for Cystectomy (DAD) | 1 | 5 | 3 | 3 | 3 |
| Diagnostic Code for Bladder Cancer (DAD) | 4 | 3 | 3.5 | 4 | 4 |
| Urology was Main Provider Service (DAD) | 5 | 4 | 4.5 | 5 | 5 |
| Operative Time (DAD)* | 6 | 7 | 6.5 | 6 | 9 |
| Operation done in main OR (DAD) | 11 | 6 | 8.5 | 7 | 10 |
| Acute Length of Stay (DAD) | 7 | 11 | 9 | 8 | 14 |
| Patient Age (DAD)* | 8 | 10 | 9 | 9 | 18 |
| Elective Admission (DAD) | 10 | 9 | 9.5 | 10 | 19 |
| Anesthetic Type (DAD) | 12 | 8 | 10 | 11 | 20 |
| Readmission or Death within 28 days (DAD) | 9 | 13 | 11 | 12 | 21 |
| Blood Transfusion (DAD) | 14 | 12 | 13 | 13 | 22 |
| Unplanned Return to the OR (DAD) | 13 | 14 | 13.5 | 14 | 23 |

OHIP = Ontario Health Insurance Plan; OCR = Ontario Cancer Registry; DAD = Discharge Abstract Database; OR = operating room. * = continuous variable consuming 4 degrees of freedom (2 df for 2 fractional polynomial terms and 2 df for optimal polynomial identifiecation); all other variables consumed 1 degree of freedom.

**APPENDIX D:** The Administrative Database Cystectomy Models, by Diversion type.

| **VARIABLE** | **Cystectomy-Incontinent Diversion** | | **Cystectomy-Continent Diversion** | |
| --- | --- | --- | --- | --- |
|  | **Parameter Estimate (95%CI)** | **Adjusted Odds Ratio (95% CI)** | **Parameter Estimate (95% CI)** | **Adjusted Odds Ratio (95% CI)** |
| Intercept | -10.97 | - | -26.64 | - |
| Bladder Cancer - DAD | -0.03 (-1.08, 1.02) | 0.97 (0.34, 2.8) | 2.40 (0.62, 4.17) | 11.0 (1.9, 64.8) |
| Elective Admission | 1.66 (0.63, 2.67) | 5.3 (1.88, 14.8) | 2.53 (0.52, 4.54) | 12.5 (1.7, 93.3) |
| Urology is primary Service | 6.58 (4.86, 8.31) | 722.6 (128.9, >999.9) | 14.27 (-840.4, 868.9) | >999.9 (<0.001, >999.9) |
| Cystectomy Code - Incontinent: OHIP | 3.66 (2.53, 4.79) | 38.8 (12.5, 120.6) | 0.22 (-1.13, 1.57) | 1.3 (0.32, 4.8) |
| CCI | 5.87 (4.68, 7.06) | 352.8 (107.3, >999.9) | 3.65 (0.83, 6.46) | 38.4 (2.3, 640.7) |
| - Continent: OHIP | 0.67 (-0.48, 1.81) | 2.0 (0.62, 6.1) | 7.16 (4.82, 9.50) | >999.9 (123.7, >999.9) |
| CCI | 4.85 (3.67, 6.04) | 129.2 (39.2, 419.7) | 6.21 (3.74, 8.68) | 498.5 (42.0, >999.9) |
| Unplanned Return to OR in 28d | 1.99 (-0.17, 4.14) | 7.3 (0.84, 63.1) | 3.99 (1.18, 6.80) | 54.2 (3.3, 901.5) |
| Acute LOS | -0.05 (-0.08, -0.02) | 0.95 (0.92, 0.98) | -0.03 (-0.07, 0.008) | 0.97 (0.93, 1.01) |
| 1/(Acute LOS)^0.5^ | -9.37 (-14.4, -4.32) | <0.001 (<0.001, 0.013) | -4.59 (-14.7, 5.49) | 0.01 (<0.001, 241.2) |

To measure the expected probability of either Cystectomy-Incontinent Diversion or Cystectomy-Continent Diversion for a particular patient, one needs to calculate the linear predictor (LP). This is done by first determining that person’s value for all variables in the model (Column 1); specific code values are given in Appendix B. LP is sum of the intercept and parameter estimates * variable value. Expected probability is e^LP^/(1+e^LP^).

DAD = Discharge Abstract Database; OHIP = Ontario Health Insurance Plan; CCI = Canadian Codes for Interventions; LOS = length of

#### APPENDIX E: TRIPOD (Transparent reporting of a multivariable prediction model for individual prognosis or diagnosis) Checklist for the Administrative Database Cystectomy Models

| **Section/Topic** | **Item** | **Checklist Item** | **Page** |
| --- | --- | --- | --- |
| **Title and abstract** | | | |
| Title | 1 | Identify the study as developing and/or validating a multivariable prediction model, the target population, and the outcome to be predicted. | 1 |
| Abstract | 2 | Provide a summary of objectives, study design, setting, participants, sample size, predictors, outcome, statistical analysis, results, and conclusions. | 2 |
| **Introduction** | | | |
| Background and objectives | 3a | Explain the medical context (including whether diagnostic or prognostic) and rationale for developing or validating the multivariable prediction model, including references to existing models. | 4 |
|  | 3b | Specify the objectives, including whether the study describes the development or validation of the model or both. | 4 |
| **Methods** | | | |
| Source of data | 4a | Describe the study design or source of data (e.g., randomized trial, cohort, or registry data), separately for the development and validation data sets, if applicable. | 6 |
|  | 4b | Specify the key study dates, including start of accrual; end of accrual; and, if applicable, end of follow-up. | 6 |
| Participants | 5a | Specify key elements of the study setting (e.g., primary care, secondary care, general population) including number and location of centres. | 5 |
|  | 5b | Describe eligibility criteria for participants. | 7 |
|  | 5c | Give details of treatments received, if relevant. | NA |
| Outcome | 6a | Clearly define the outcome that is predicted by the prediction model, including how and when assessed. | 8 |
|  | 6b | Report any actions to blind assessment of the outcome to be predicted. | 7 |
| Predictors | 7a | Clearly define all predictors used in developing or validating the multivariable prediction model, including how and when they were measured. | APP. C |
|  | 7b | Report any actions to blind assessment of predictors for the outcome and other predictors. | NA |
| Sample size | 8 | Explain how the study size was arrived at. | FIG 1 |
| Missing data | 9 | Describe how missing data were handled (e.g., complete-case analysis, single imputation, multiple imputation) with details of any imputation method. | NA |
| Statistical analysis methods | 10a | Describe how predictors were handled in the analyses. | 8 |
|  | 10b | Specify type of model, all model-building procedures (including any predictor selection), and method for internal validation. | 7 |
|  | 10d | Specify all measures used to assess model performance and, if relevant, to compare multiple models. | 8-9 |
| Risk groups | 11 | Provide details on how risk groups were created, if done. | NA |
| **Results** | | | |
| Participants | 13a | Describe the flow of participants through the study, including the number of participants with and without the outcome and, if applicable, a summary of the follow-up time. A diagram may be helpful. | FIG 1 |
|  | 13b | Describe the characteristics of the participants (basic demographics, clinical features, available predictors), including the number of participants with missing data for predictors and outcome. | TABLE 2 |
| Model development | 14a | Specify the number of participants and outcome events in each analysis. | 11 |
|  | 14b | If done, report the unadjusted association between each candidate predictor and outcome. | APP. D |
| Model specification | 15a | Present the full prediction model to allow predictions for individuals (i.e., all regression coefficients, and model intercept or baseline survival at a given time point). | APP. D |
|  | 15b | Explain how to the use the prediction model. | APP. D |
| Model performance | 16 | Report performance measures (with CIs) for the prediction model. | 11-12 |
| **Discussion** | | | |
| Limitations | 18 | Discuss any limitations of the study (such as nonrepresentative sample, few events per predictor, missing data). | 14 |
| Interpretation | 19b | Give an overall interpretation of the results, considering objectives, limitations, and results from similar studies, and other relevant evidence. | 13 |
| Implications | 20 | Discuss the potential clinical use of the model and implications for future research. | 14 |
| **Other information** | | | |
| Supplementary information | 21 | Provide information about the availability of supplementary resources, such as study protocol, Web calculator, and data sets. | NA |
| Funding | 22 | Give the source of funding and the role of the funders for the present study. | 15 |

We recommend using the TRIPOD Checklist in conjunction with the TRIPOD Explanation and Elaboration document.
